# All-Purpose Measure of Electron Correlation for Multireference Diagnostics

**DOI:** 10.1021/acs.jctc.3c01073

**Published:** 2023-12-29

**Authors:** Xiang Xu, Luis Soriano-Agueda, Xabier López, Eloy Ramos-Cordoba, Eduard Matito

**Affiliations:** † Donostia International Physics Center (DIPC), 20018 Donostia, Euskadi, Spain; ‡ Polimero eta Material Aurreratuak: Fisika, Kimika eta Teknologia, Kimika Fakultatea, Euskal Herriko Unibertsitatea UPV/EHU, P.K. 1072, 20080 Donostia, Euskadi, Spain; § Ikerbasque Foundation for Science, Plaza Euskadi 5, 48009 Bilbao, Spain

## Abstract

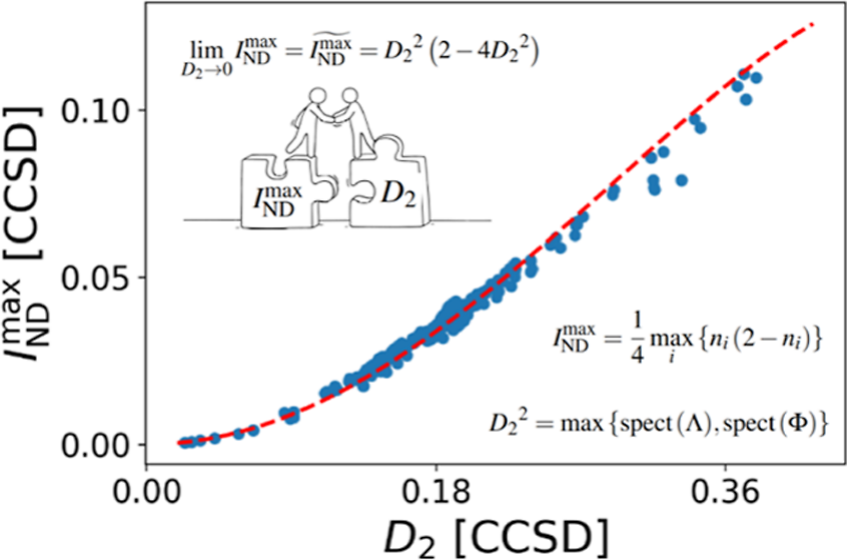

We present an analytical relationship between two natural orbital occupancy-based indices,  and *I*_ND_^max^, and two established electron correlation metrics: the leading term of a configuration interaction expansion, *c*_0_, and the *D*_2_ diagnostic. Numerical validation revealed that  and *I*_ND_^max^ can effectively substitute for *c*_0_ and *D*_2_, respectively. These indices offer three distinct advantages: (i) they are universally applicable across all electronic structure methods, (ii) their interpretation is more intuitive, and (iii) they can be readily incorporated into the development of hybrid electronic structure methods. Additionally, we draw a distinction between correlation measures and correlation diagnostics, establishing MP2 and CCSD numerical thresholds for *I*_ND_^max^, which are to be used as a multireference diagnostic. Our findings further demonstrate that establishing thresholds for other electronic structure methods can be easily accomplished using small data sets.

## Introduction

Statistical learning has been recently applied to increase the predictability of quantum chemistry methods for various purposes.^1−3^ To effectively train artificial neural networks for various tasks, such as image recognition, natural language processing, and drug discovery, large-scale screening of extensive data sets is essential. Since molecular systems with a large multireference (MR) character compromise the performance of cost-effective methods such as density functional approximations,^4^ there is a need for MR diagnostics that can be universally applied to different electronic structure methods.^5−7^

Insights into the MR character of a molecule require knowledge of the exact wave function and an uncorrelated counterpart. The distance between these wave functions is assessed using a specific metric, giving rise to a correlation measure. There is an undeniable arbitrariness in the choice of the metric, giving rise to many possible correlation measures.^8^ However, some reasonable restrictions might be imposed to constrain the set of valid measures. Bartlett et al. logically impose size-intensiveness to make the measures comparable among systems of different sizes.^9^ Furthermore, a pragmatic approach suggests the development of measures that can be easily implemented across various electronic structure methods. Beyond these two prerequisites, it is desirable for these quantities to be easily incorporated in the development of new electronic structure methods.^10−62^ As an example, correlation measures derived from natural orbital occupancies have been instrumental in the evolution of hybrid electronic structure methodologies that seamlessly integrate diverse correlation approaches.^14−17^ Nevertheless, the primary and most common application of correlation measures remains their role as MR diagnostics.

In our perspective, MR diagnostics bring forth an additional criterion for correlation measures: the capacity to capture the most profoundly correlated aspects of the system. This principle holds true regardless of the system’s size; even if only a small fraction of it exhibits features necessitating a MR description, it justifies the classification of the molecule as MR. In this context, it is only logical to employ correlation measures founded on the concept of maximal distance between wave functions. While this criterion does introduce additional constraints on correlation measures, it still allows for a considerable degree of flexibility. The *T*_1_ diagnostic^18^ relies on the Frobenius norm of *t*_1_ coupled-cluster amplitudes, representing an average value. In contrast, *D*_1_(19) and *D*_2_(20) diagnostics are grounded in the 2-norm of matrices constructed from the *t*_1_-amplitude and *t*_2_-amplitude tensors, respectively. These norms are associated with the maximum eigenvalues of these matrices and are better suited to MR diagnostics. However, one must heed the warning that measures, like *D*_1_, which are based on single excitations, are susceptible to orbital rotation and thus should be avoided.^21−23^

In practice, the correlation measure requires an approximate wave function—that introduces electron correlation to some extent but is far from the exact wave function—and an uncorrelated reference such as the Hartree–Fock (HF) wave function. HF is the most natural choice for wave function methods. The HF wave function corresponds to a single Slater determinant including the HF orbitals that are obtained from a variational minimization of the electronic energy subject to the orthonormality constraint of the orbitals. The resulting HF electron density presents a simple diagonal representation in terms of the HF canonical orbitals, where some orbitals are occupied (with an occupation of one electron each), while others are regarded as virtual and do not contribute to the electron density (occupation equal to zero). In general, post-HF wave functions do not admit a diagonal representation of the electron density in terms of HF orbitals, the orbitals providing a diagonal representation of the electron density being the natural orbitals.^24^ Essentially, HF orbitals can be regarded as the natural orbitals of the HF wave function, the occupation of which is fixed by their energy and the number of electrons in the system. Natural orbital occupancies of post-HF wave functions are generally noninteger, the deviation from the closest integer number, 0 or 1, providing a straightforward way to assess the effect of electron correlation in the density. In this sense, measures of electron correlation based on natural orbital occupancies (NOOs)^9,23−31^ are a most convenient way of measuring the effects of electron correlation as they only require the natural orbital representation of the electron density of the target method.

In a recent investigation,^6^ Kulik and colleagues leaned on the NOO indicators developed in our group.^30,31^ These indicators were favored due to their ability to exhibit robust linear correlations, achieving a Pearson coefficient of 65% with energy-based correlation metrics like the contribution of perturbative triples to the CCSD(T) correlation energy, %*E*_corr_[(T)]. This study suggests that indicators like *D*_2_ (75%) might offer a more accurate reflection of the MR character; however, they are typically computed only for coupled-cluster wave functions. While it is possible to calculate *D*_2_ using MP2, it is not a common practice.

In this article, we propose a NOO correlation measure that meets the aforementioned conditions and can thus be employed as a MR diagnostic. We show that this quantity is connected to *D*_2_, however, unlike the latter, it can be straightforwardly used for all types of electronic structure methods, provided NOOs are available. In this regard, it can be even employed for density functional approximations as fractional occupancies can be derived from Kohn–Sham orbital energies^7,29,32^ or unrestricted Kohn–Sham NOOs.^33^ Similarly, the latter and other NOO correlation measures find a natural framework within ensemble DFT,^34^ thermally assisted occupation DFT,^35^ and the first-order reduced density matrix functional theory.^36−38^

## Methodology

Some of us have recently defined indices of dynamic and nondynamic correlation based on NOOs,^30,31^ rooted on the Lieb partition^39−41^ of the pair density^42,43^
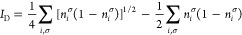
1
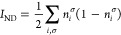
2where *n*_*i*_^σ^ stands for the *i*th natural orbital occupation with spin σ. For convenience, we assume natural orbitals are arranged in descending order based on their occupation. *I*_ND_ coincides with the deviation from idempotency of the first-order reduced density matrix,^24,25^ takes values in the interval [0, *N*/2], and for closed-shell systems can be recasted as
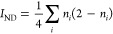
3where *n*_*i*_ now takes values between zero and two. All these quantities are size-extensive, as noted by some authors,^44^ and *I*_ND_ can be easily converted to a size-intensive one
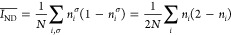
4where *N* stands for the number of electrons and the last equality only holds for closed-shell systems. Sometimes it is convenient to split  into *pseudo-occupied* (corresponding to the *N*^α^ and *N*^β^ highest occupied) and the *pseudo-virtual* (the rest) spin natural orbitals
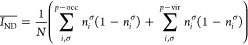
5and as other authors have found,^9^ we have heuristically identified that
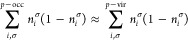
6which reflects the symmetric nature of particles and holes often encountered in the first-order reduced density matrix of molecular systems (see Figure S1 for a convincing numerical test). Hence
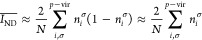
7

From , we can construct the maximum counterpart
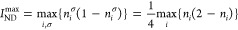
8the latter equality being valid only for closed-shell molecules. One can easily prove that  and . However, these maximal values are reached for very different situations. The maximal value of *I*_ND_^max^ is achieved for ; consequently, we only require one natural orbital with an occupation midway between being occupied and empty. On the other hand, the supremum of  is approached when we have a very large number of orbitals relative to the number of electrons, all of which attain a very small occupation value (∀*i*, *n*_*i*_ = *N*/*M* ∧ *M* → ∞, *M* being the number of orbitals). This situation requires an extremely correlated regime only achieved in very peculiar model systems.^13,38^ In practice, for regular molecular systems, large  values are usually obtained because one or various occupation numbers deviate significantly from 0 and 1 (hence, they are somewhat close to ) contributing to a large value relative to the total number of electrons. Since *I*_D_ is not bounded above, its size-intensive version becomes complicated and necessarily uses a different normalization than . However, for practical purposes, we suggest employing , in analogy to . In Figures S14 and S15 in Supporting Information, we demonstrate that  has a small sensitivity to the basis set, whereas  varies much more significantly with the quality of the basis set as expected from dynamic correlation measures.

In the following, we will show how  and *I*_ND_^max^ can be *analytically* connected to other existing measures of electron correlation. To this end, we will first consider two approximate wave functions: a configuration interaction singles and doubles (CISD) and a closed-shell MP2 wave function. Finally, the relationships found for these methods will be numerically tested for other wave function methods to assess their general validity.

### Connection between the CI Leading Expansion Coefficient and 

Let us assume a CISD wave function expansion
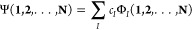
9where we have used the short-hand notation  to indicate the spatial and spin coordinates of electron 1. Φ_I_ corresponds to Slater determinants constructed from HF orbitals, and *c*_I_ are the expansion coefficients, *c*_0_ being the one associated with the HF Slater determinant and assumed to be the dominant one. Without loss of generality, Ψ is normalized to 1. For convenience, we define the following quantities
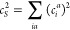
10
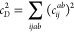
11where *c*_*i*_^*a*^ and *c*_*ij*_^*ab*^ represent the coefficient expansions for single and double excitations, respectively. These expansions are associated with the Slater determinants formed by substituting orbital *i* with orbital *a* and orbitals (*i*, *j*) with orbitals (*a*, *b*). *i*, *j* and *a*, *b* are occupied and virtual orbitals in the HF wave function, respectively.

Hence, we can write
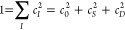
12

**Figure 1:**
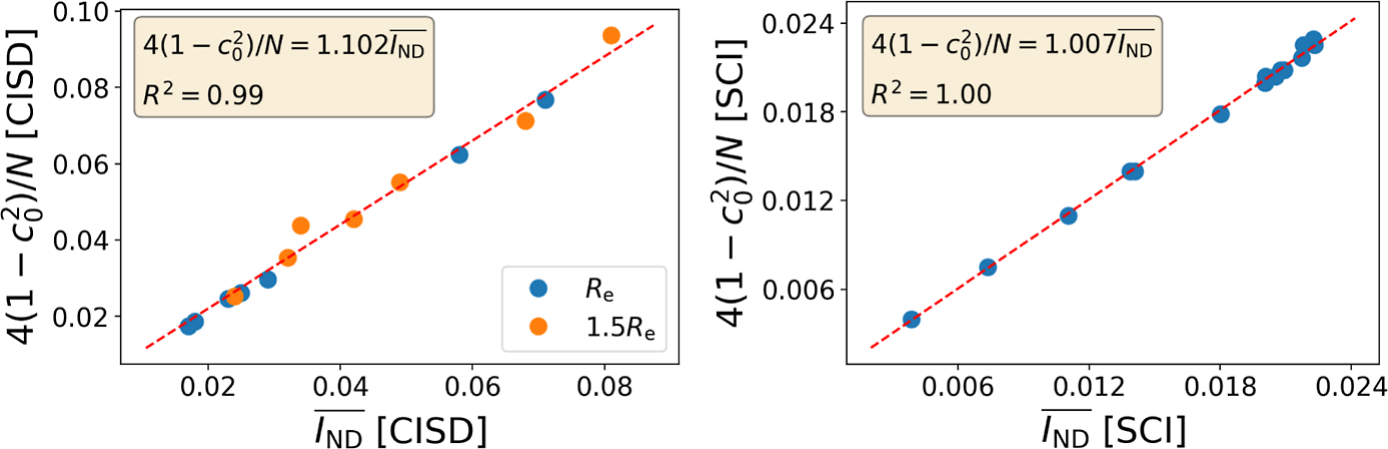
Relationship between 4(1-*c*_0_^2^)/*N* and  for (left) Set A, including various diatomic molecules at equilibrium and 50% larger interatomic distances at the CISD level, and (right) Set B containing several 18-electron systems studied at the selected CI (SCI) level.

Let us consider the electron density constructed from this wave function
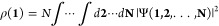
13which can be expanded in terms of the HF molecular orbitals, {ϕ_*i*_(**1**)}
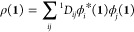
14with
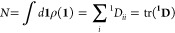
15We can decompose the trace of ^1^**D** into two parts, corresponding to the submatrices we can construct from the orbitals that are occupied and empty in the HF picture
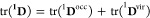
16which can be calculated from
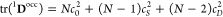
17
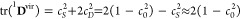
18where we have assumed that the role of single excitations is significantly smaller than twice of all the excitations. For a dominant block-diagonal ^1^**D** matrix, as expected for nonhighly correlated systems, we have
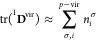
19From the latter two approximations and eq 7, we find
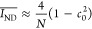
20connecting the leading term in the CI expansion with a natural-orbital-based measure of electron correlation. In Figure 1, we can numerically confirm this relationship for various diatomic molecules at equilibrium and larger interatomic distances, as well as a collection of 18-electron systems. Notice that both CISD and a selected CI expansion give excellent agreement. For the same token, it becomes evident that (1-*c*_0_^2^) should exhibit a proportional relationship with *I*_ND_ and, considering the latter is not size-intensive, it follows that *c*_0_^2^ also lacks this property.

Some of us had recently^42^ suggested a size-intensive version of *c*_0_, 1-*c*_0_^2/*N*^, which adopts the following Taylor series around *c*_0_^2^ → 1
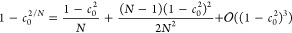
21

Interestingly, the leading term in the size-intensive version of *c*_0_ is consistent with the approximation obtained for  in eq 20 as is numerically confirmed in Figure S12. These results strongly suggest that, notwithstanding the well-founded criticisms raised in previous studies,^45−47^ with appropriate normalization, *c*_0_ can serve as a reliable correlation measure. In practical applications, the use of  is recommended over *c*_0_ because it is not limited to CI expansions.

### Connection between *D*_2_ Diagnostic and *I*_ND_^max^

The unrelaxed MP2 electron density up to the second-order correction in terms of canonical HF doubly occupied orbitals reads^48,49^
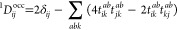
22
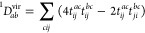
23
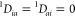
24where *i*, *j* and *a*, *b* denote *occupied* and *virtual* orbitals, respectively, *t*_*ij*_^*ab*^ being first-order corrections to double amplitudes.^49^ For convenience, let us define
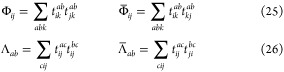
25and identify *n*_*H*_ and *n*_*L*_ as the lowest and highest eigenvalues of ^1^**D**^occ^ and ^1^**D**^vir^, i.e., *n*_*H*_ = min_*i*≤*N*/2_{*n*_*i*_} and *n*_*L*_ = max_*i*>*N*/2_{*n*_*i*_}
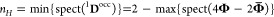
27
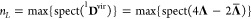
28*n*_*i*_ being the NOOs for the closed-shell unrelaxed MP2 density matrix arranged in descending order.

If we disregard the last term in eqs 22 and 23, we construct an approximate ^1^**D**, containing these blocks
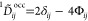
29
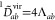
30that bring approximate counterparts of eqs 27 and 28
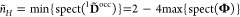
31
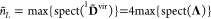
32which can be connected with *n*_*H*_ and *n*_*L*_(50)
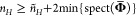
33
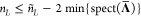
34Since  and  are semipositive defined,  and . Hence,  and  overestimate electron correlation. In Figures S8–S11, it can be checked that this overestimation grows with electron correlation.

Nielsen and Janssen^20^ defined the *D*_2_ diagnostic for closed-shell molecules as
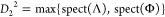
35therefore
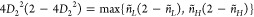
36

**Figure 2:**
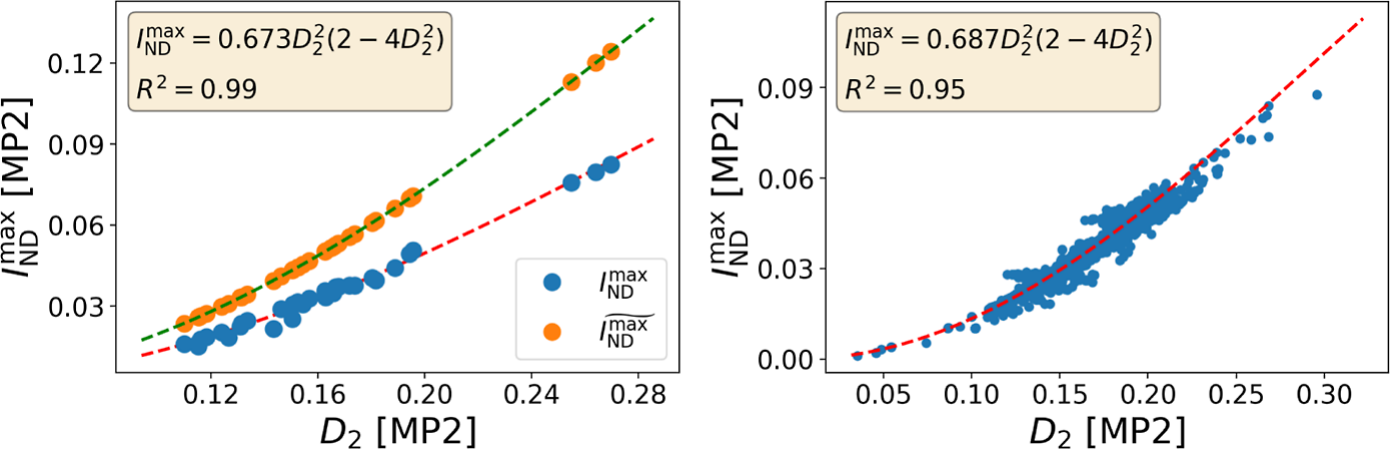
*I*_ND_^max^ and  against *D*_2_ for the molecules in Set C (left) and *I*_ND_^max^ against *D*_2_ for the molecules in Set D (right). All calculations were performed at the MP2 level.

**Figure 3:**
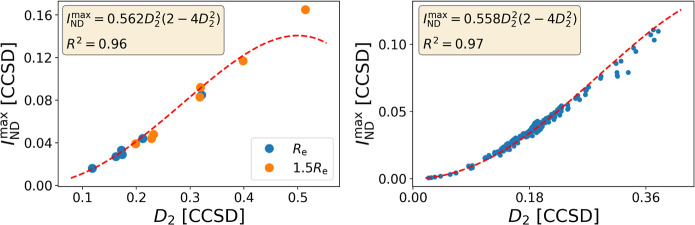
Relationship between *D*_2_ and *I*_ND_^max^ for Set A (left) and Set E (right) at the CCSD level.

On the other hand, we can rewrite eq 8 as
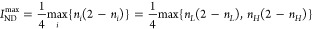
37which we can now connect to *D*_2_
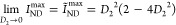
38 for an unrelaxed MP2 electron density including up to second-order corrections in which we have neglected some terms. In practice, the latter terms contribute to the electron density and thus affect the value of natural occupancies. In Figure 2, we can identify the effect of this approximation. Since  and  overestimate electron correlation, we know that systematically . Indeed, we find good correspondence between *D*_2_ and *I*_ND_^max^ for MP2 calculations (see Figure 2 for a large-set validation). As we can see in Figure 3, the relationship is not limited to MP2, giving a very good correlation for CCSD too. A separate analysis of the HEAVY28 and HEAVYSB11 data sets (included in GMTKN55,^51^ which is part of Set D) reveals that this connection holds also for transition-metal complexes (see Figure S13), hence the transferability issues for diagnostics like *T*_1_ are not of concern in the present context.^52^

The strong correlation between *I*_ND_^max^ and *D*_2_ starts to diminish at large values of *D*_2_, well above the recommended MR diagnostic thresholds (*D*_2_ > 0.15–0.18). We can therefore interchangeably utilize either *D*_2_ or *I*_ND_^max^ in the design of an MR diagnostic. Since *I*_ND_^max^ can be employed for all sorts of wave functions, its use is recommended over *D*_2_, which can be only computed for MP2 and CC, but in practice is often limited to CC.

Nielsen and Janssen^20^ set *D*_2_ = 0.15 as the boundary below which is safe to trust MP2 and CCSD results, hence the molecule can be classified as non-MR. Conversely, *D*_2_ ≥ 0.17 (MP2) and *D*_2_ ≥ 0.18 (CCSD) signal the molecule as MR. In between these values, the molecule should be considered with caution. Based on the fittings of *I*_ND_^max^ against *D*_2_ for sets A, C, D, and E (see Figures 2 and 3), we find an equivalence with the *D*_2_ thresholds established by Nielsen and Janssen (see Table 1).^20^ For the same method, the MR diagnostic thresholds of *I*_ND_^max^ barely change regardless of whether we use a modest data set like set A and C or whether we employ much larger ones such as D or E, setting some confidence on the connection between both sets of thresholds. In all cases, the agreement between the diagnostics (the percentage of molecules equally classified as MR or non-MR) is above 94%, validating the replacement of *D*_2_ by *I*_ND_^max^.

**Table 1:** Equivalence between *D*_2_ and *I*_ND_^max^ Thresholds for MR Diagnostics

method	set	size	*D*_2_	*I*_ND_^max^	agreement(%)
MP2	set C	34	0.15	0.029	94
MP2	set C	34	0.17	0.037	97
MP2	set D	5090	0.15	0.030	98
MP2	set D	5090	0.17	0.037	96
CCSD	set A	14	0.15	0.024	100
CCSD	set A	14	0.18	0.034	100
CCSD	set E	311	0.15	0.024	95
CCSD	set E	311	0.18	0.034	97

## Conclusions

In this study, we presented the analytical relationship between two NOO-based indices and two established electron correlation metrics: the leading term of the CI expansion, *c*_0_, and the *D*_2_ diagnostic. Numerical validation revealed that  and *I*_ND_^max^, which are straightforwardly connected to the deviation from idempotency of the first-order reduced density matrix, can effectively substitute for *c*_0_ and *D*_2_, respectively.

Utilizing  and *I*_ND_^max^ offers three distinct advantages:

1. They are universally applicable across all electronic structure methods, as long as NOOs are accessible.
2. Their interpretation is more intuitive, given that they correspond to the *average* and the *maximal* deviation of NOOs from zero and one, respectively. Notably, juxtaposing both indices offers a comprehensive evaluation of electron correlation within a molecule, but only *I*_ND_^max^ is recommended as MR diagnostic.
3. As they solely depend on NOOs, these indices can be effortlessly incorporated into the development of hybrid electronic structure methods.

Furthermore, we have furnished numerical thresholds for *I*_ND_^max^ pertinent to MP2 and CCSD wave functions, facilitating its use as MR diagnostic. Owing to the consistent alignment between *D*_2_ and *I*_ND_^max^ thresholds across different data sets, deriving thresholds for alternative electronic structure methods using compact test sets, such as A and C, becomes straightforward.

## Computational Details

All CISD and CCSD calculations and geometry optimizations were carried out with Psi4,^53−55^ whereas all MP2 and Selected Configuration Interaction (SCI) calculations^56,57^ were carried out with PySCF.^58,59^ The SCI values should be fairly close to those of the FCI solution. To effectively establish a connection between correlation measures, it is not essential to focus primarily on the nature of the data sets. Instead, our approach emphasizes the selection of tests that encompass a diverse range of correlation regimes and a wide variety of molecules in our study. In particular, we considered the following test sets:

- Set A: Seven small diatomic molecules at the equilibrium distance (*R*_e_) and stretched geometry (1.5 *R*_e_) studied by Handy et al.^60^ at the CISD/cc-pVTZ and CCSD/cc-pVTZ levels of theory.
- Set B: Crittenden and Gill’s^61^ 18-electron systems extended with other such molecules. The geometries were optimized at the MP2/6-31G* level, and single-point calculations were performed at the CISD/6-311G and SCI/6-311G levels of theory. This reduced basis set was employed due to the SCI computational cost.
- Set C: 34 small molecules of Nielsen and Janssen^20^ from which they defined the *D*_1_ and *D*_2_ diagnostics. Geometries and single-point calculations were performed at the MP2/cc-pVTZ level of theory.
- Set D: A 5090 closed-shell molecular set obtained from merging part of the GMTKN55 data set^51^ and the AD-3165 data set.^7^ The former was included in order to cover the spectrum of low-correlated molecules, which was undersampled with the AD-3165 data set. From GMTKN55, we considered 1925 closed-shell molecules among the total 2462 molecular structures, excluding molecules that carried a considerable computational cost. Calculations were done at the MP2/def2-qzvp level. From the AD-3165 data set of Kulik and co-workers,^7^ we extracted geometries and performed the MP2/cc-pVTZ calculations.
- Set E: A 311-molecule diverse set with *D*_2_ ≤ 0.4 computed at the CCSD level. See the Supporting Information for the detailed list of molecules.

## Supporting Information Available

Computational details, figures, and basis set dependence (PDF)
List of molecules excluded from the GMTKN55 dataset (XLSX)

## Notes

Further computational details can be found in the Supporting Information.
