# Supplementary material for: All-Purpose Measure of Electron Correlation for Multireference Diagnostics
Source: J Chem Theory Comput. 2023 Dec 29;20(2):721–7. doi: 10.1021/acs.jctc.3c01073 (PMC10809408; doi:10.1021/acs.jctc.3c01073)
Supplement: Supplementary file 1 — ct3c01073_si_001.pdf [file ct3c01073_si_001.pdf]

Supporting Information: An All-Purpose Measure of Electron Correlation for Multireference Diagnostics

Xiang Xu,[†,‡] Luis Soriano-Agueda,[†] Xabier López,[†,‡] Eloy Ramos-Cordoba,[*,†,‡,¶] and Eduard Matito[*,†,¶]

†*Donostia International Physics Center (DIPC), 20018 Donostia, Euskadi, Spain*

‡*Polimero eta Material Aurreratuak: Fisika, Kimika eta Teknologia, Kimika Fakultatea, Euskal Herriko Unibertsitatea UPV/EHU, P.K. 1072, 20080 Donostia, Euskadi, Spain.*

¶*Ikerbasque Foundation for Science, Plaza Euskadi 5, 48009 Bilbao, Euskadi, Spain*

E-mail: eloy.raco@gmail.com; ematito@gmail.com

Computational Details

All CISD and CCSD calculations and geometry optimizations were carried out with `Psi4`,[1–3] whereas all MP2 and Selected Configuration Interaction (SCI) calculations[4,5] were carried out with `PySCF`.[6,7] The SCI values should be fairly close to the FCI solution. All the post-HF calculations employed frozen core (except for the 18-electron systems, i.e., **Set B**), and the energy convergence threshold was set to 10^{-8}. We considered the following test sets:

- **Set A**: 7 small diatomic molecules at the equilibrium distance (R_e) and stretched geometry ($1.5R_e$) that have been studied by Handy et al.[8] and in our previous study on correlation measures.[9] The cc-pVTZ basis set[10] was employed.

- **Set B**: Extended Crittenden and Gill's eighteen-electron systems:[11] Ar, C_2H_6, N_2H_4, CH_3OH, F_2, H_2O_2, H_2S, HCl, PH_3, and SiH_4 (Crittenden and Gill's set); CH_3F, CH_3NH_2, HOF, HOOH, NH_2F, and NH_2OH (extended molecule subset). The geometries were optimized at the MP2/6-31G* level, and single-point calculations were performed at the CISD/6-311G and SCI/6-311G levels of theory. This reduced basis set was employed due to the SCI computational cost.

- **Set C**: 34 small molecules of Nielsen and Janssen[12] from which they defined the D_1 and D_2 diagnostics. Geometries and single-point calculations were performed at the MP2/cc-pVTZ level of theory.

- **Set D**: A 5090 closed-shell molecular set obtained from merging part of the GMTKN55 dataset[13] and the AD-3165 dataset.[14] The former was included in order to cover the spectrum of low-correlated molecules, which was undersampled with the AD-3165 dataset. From GMTKN55, we considered 1925 closed-shell molecules among the total 2462 molecular structures, excluding molecules that carried a large computational cost. For the full table of excluded molecular structures see the file "537mol-excluded.xlsx" in the supplementary material. Calculations were done at the MP2/def2-qzvp level. For the WATER27 subset, the basis set was increased with diffuse functions *s* and *p* for the oxygen atoms. In addition, in subsets G21EA, AHB21, and IL16, the diffuse functions *s* and *p* were added for all non-hydrogen atoms, and the diffuse functions *s* were added to the hydrogen atom. The core-electron of heavy elements in some systems of HEAVY28 (Pb, Sb, Te, Bi, I), HEAVYSB11 (Pb, Sn, Te) and HAL59 (I) were replaced by the def2-ECP effective core potentials.[13] From the AD-3165 dataset of Kulik and coworkers,[14] we extracted geometries and performed the MP2/cc-pVTZ calculations.

- **Set E**: 311 diverse molecular structures at the CCSD level with $D_2 \leq 0.4$ within these subsets:

  - Strong correlated systems:

* Hydrogen chains (H_{2n}) with varying chain lengths, where $n = 1,2,3,...,12$. The inter-H_2 unit distance was set to 3 Bohr, and the intra-unit distance was set to 2 Bohr. These calculations were performed using the aug-cc-pVDZ basis set.[15,16]
* Polyacetylene (PA) chains (C_nH_{n+2}) with varying chain lengths, where n took on values of 2, 4, 6, 8, and 10. The bond distances were adapted as follows: intra-unit $r_{CC} = 1.34$ Å, $r_{CH} = 1.09$ Å, and $\theta_{CCH} = 121.5°$, inter-unit $r_{CC} = 1.45$ Å. The calculations were performed using the Sadlej-pVTZ basis set.[17]
* Twisted ethylene (C_2H_4) structures,[18] the twisted angles ranging from 0 to 85° with an increment step of 5°. The aug-cc-pVTZ basis set[15,16] was employed, and the geometries were adopted Ref. 18.
* Stretched p-quinodimethane (PQM) structures,[18] the geometries were optimized at the B3LYP level. The both-end C-C bond lengths were systematically stretched from 1.35 to 1.9 Å with a step of 0.05 Å. These calculations utilized Pople's 6-31G* basis set, which includes a diffuse function p with an exponent of 0.0523.[18–21]

- Three diatomic molecular dissociations[22] with the aug-cc-pVDZ basis set:[15,16]
  * He_2 ($R = 3.0, 3.7, 4.4, 5.0$ Å)
  * HeNe ($R = 3.1, 3.5, 4.5, 7.0$ Å)
  * HeAr ($R = 5.0, 4.3, 5.5, 8.0$ Å).
- The isoelectronic series of He (He, Li^+, Be^{2+}, B^{3+}, C^{4+}, N^{5+}) using optimized even-tempered basis sets[23,24] from Ref. 22 using the procedure outlined in Ref. 25.
- Polyhedra of He[22] consisting of 2, 3, 4, 5, and 6 helium atoms, positioned at a distance of 10 Å from the geometric center of the polyhedra, using the 6-31G basis set.[26]
- Bartlett's 14 SR (excluding O_2 triplet) and 15 MR molecules[27†] with the cc-pVTZ basis set.[10,16,28]

[†] Due to a CCSD convergence problem with `Psi4`, we slightly modified the geometry of some molecules, and we computed N_2 at $R = 3.8$ Å, HF at $R = 3$ Å, C_2H_4 at 89°).

- The W4-17 dataset, which includes 160 closed-shell molecules,[29,30] with the cc-pVTZ basis set.[10]
- The S66 non-covalent interaction dataset[31] with the aug-cc-pVDZ basis set.[15,16]

We utilized our in-house `Python3` program for the computation of all the natural orbital-based indices, as well as the D_2 diagnostic of MP2. The program makes use of several libraries, including `Numpy`,[32] `Scipy`,[33] `Pandas`,[34,35] and `Matplotlib`.[36] Moreover, the `Matplotlib` library was employed for generating all the graphs.

The density matrices of all the methods that do not satisfy the Hellmann-Feynman theorem,[37,38] i.e., MP2, CCSD, and CISD, were *unrelaxed*. Relaxed density matrices are not N-representable and can lead to unphysical occupation numbers.[39,40] In practice, these small unphysical deviations (occupation numbers slightly above one or negative) do not change significantly correlation measures, but we preferred to avoid them. `PySCF` was used to compute the unrelaxed MP2 density matrices, whereas `Psi4` was employed to compute the CISD or CCSD counterparts.

Figures

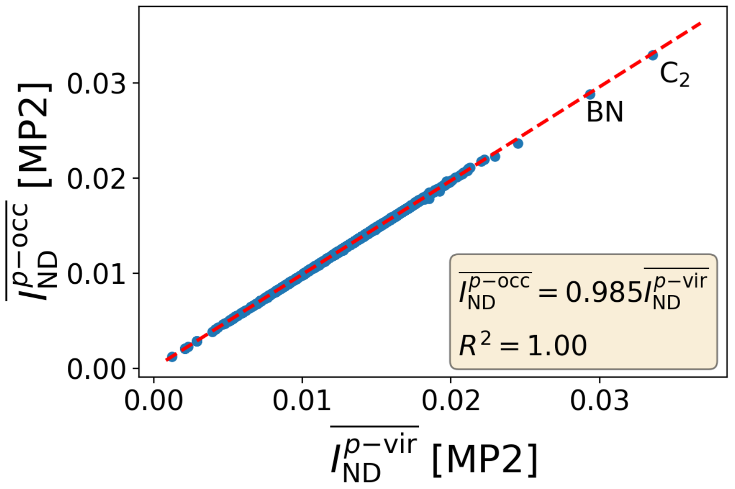

Figure S1: $\sum_{i,\sigma}^{p-\mathrm{occ}} n_i^\sigma \left(1 - n_i^\sigma\right)/N$ against $\sum_{i,\sigma}^{p-\mathrm{vir}} n_i^\sigma \left(1 - n_i^\sigma\right)/N$ for **Set D** computed at the MP2 level.

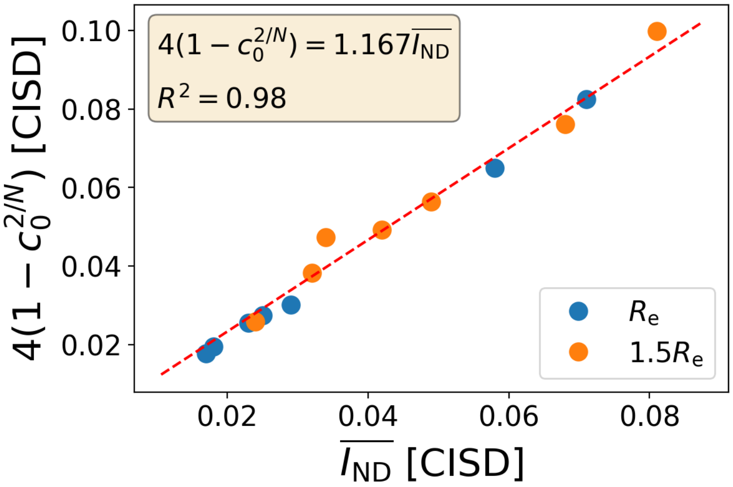

Figure S2: $1-c_0^{2/N}$ against $\overline{I_{\mathrm{ND}}}$ for **Set A** at the CISD level.

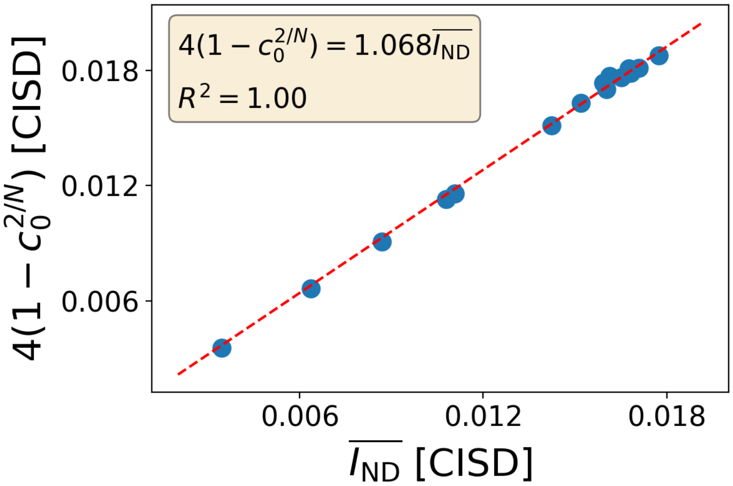

Figure S3: $1-c_0^{2/N}$ against $\overline{I_{\mathrm{ND}}}$ for **Set B** at the CISD level.

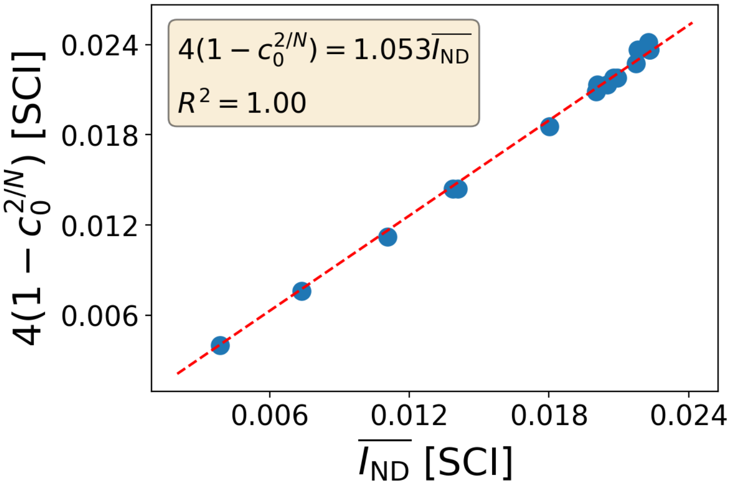

Figure S4: $1-c_0^{2/N}$ against $\overline{I_{\mathrm{ND}}}$ for **Set B** at the selected CI level.

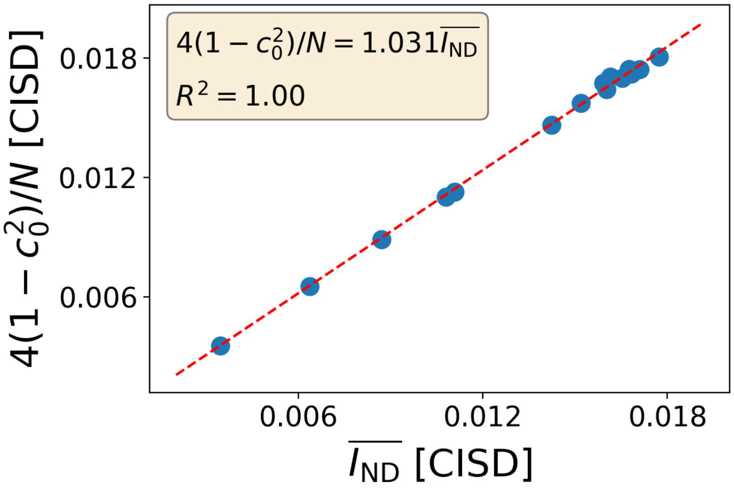

Figure S5: $4\left(1-c_0^2\right)$ against $\overline{I_{\mathrm{ND}}}$ for **Set B** at the selected CISD level.

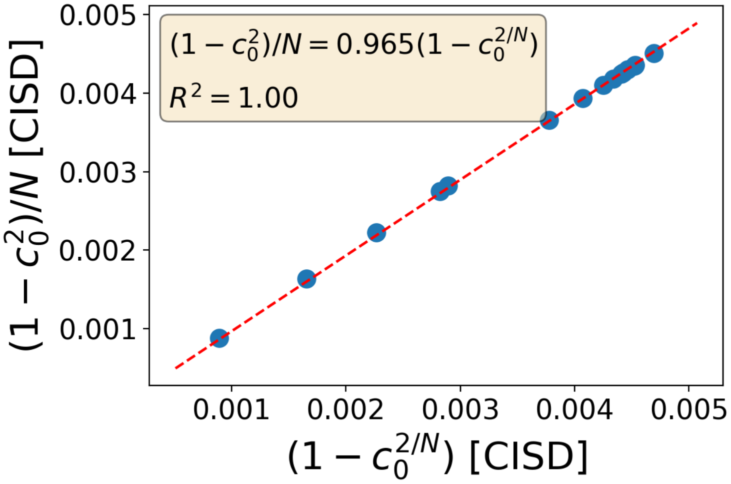

Figure S6: $\left(1-c_0^2\right)/N$ against $1-c_0^{2/N}$ for **Set B** at the CISD level.

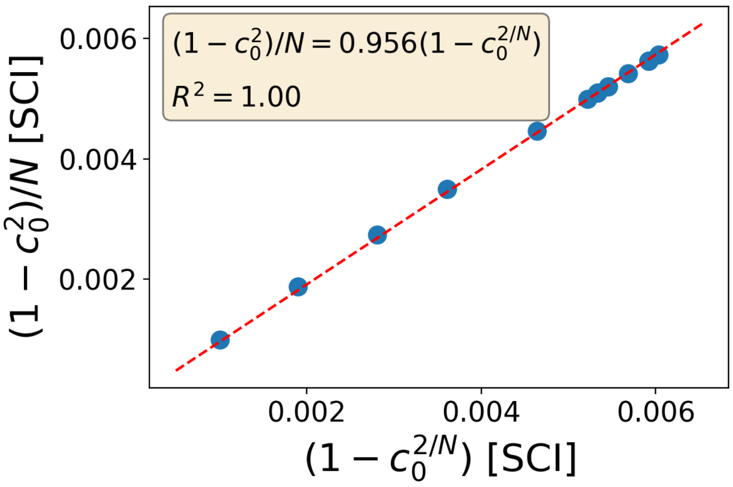

Figure S7: $\left(1-c_0^2\right)/N$ against $1-c_0^{2/N}$ for **Set B** at the selected CI level.

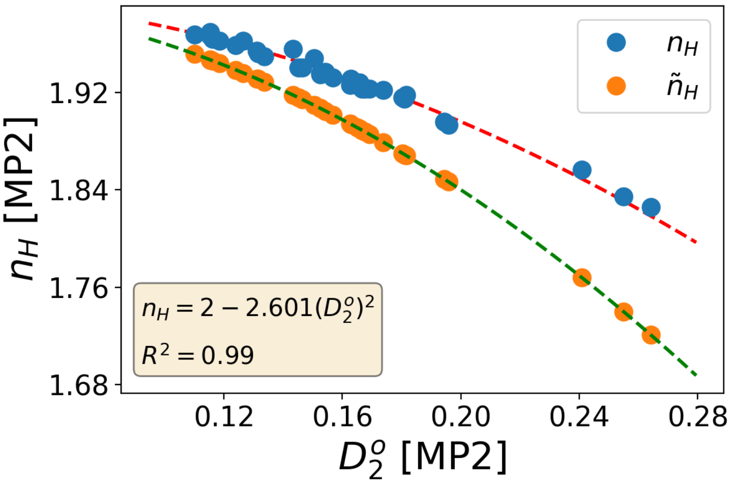

Figure S8: n_H and $\tilde{n}_H$ against D_2^o, where $(D_2^o)^2 = \max\{\mathrm{spect}(\mathbf{\Phi})\}$ for **Set C** at the MP2 level.

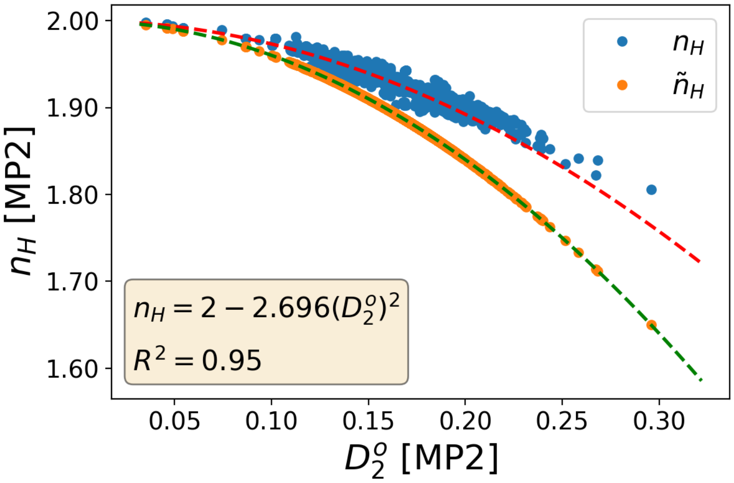

Figure S9: n_H and $\tilde{n}_H$ against D_2^o, where $(D_2^o)^2 = \max\{\mathrm{spect}(\mathbf{\Phi})\}$ for **Set D** at the MP2 level.

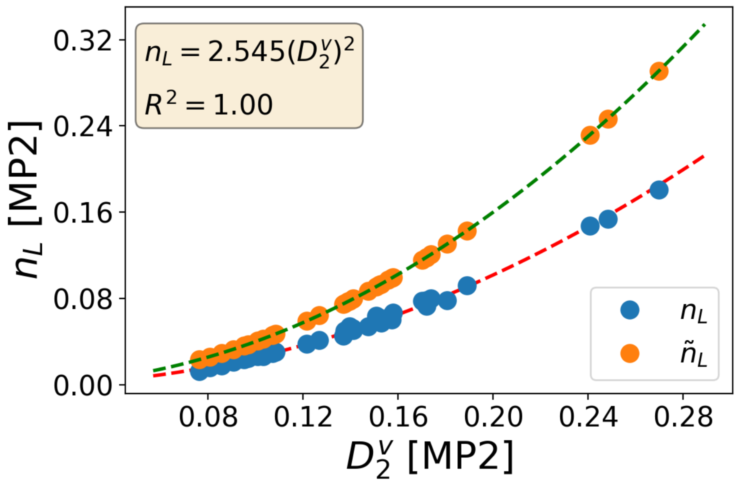

Figure S10: n_L and $\tilde{n}_L$ against D_2^v, where $(D_2^v)^2 = \max\{\text{spect}(\mathbf{\Lambda})\}$ for **Set C** at the MP2 level.

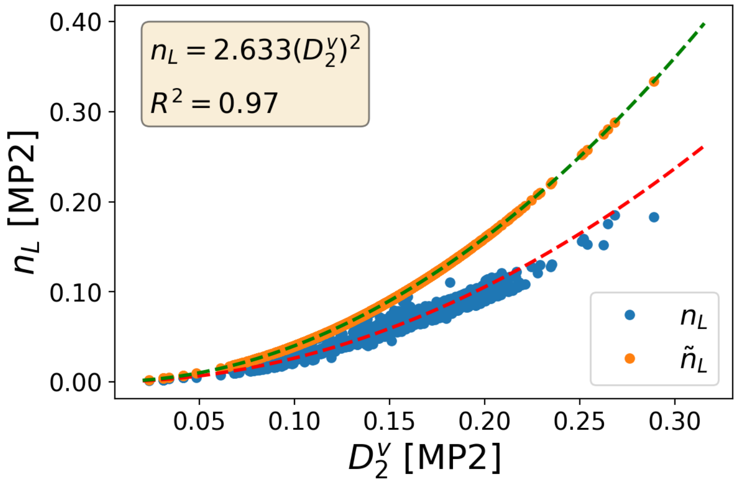

Figure S11: n_L and $\tilde{n}_L$ against D_2^v, where $(D_2^v)^2 = \max\{\text{spect}(\mathbf{\Lambda})\}$ for **Set D** at the MP2 level.

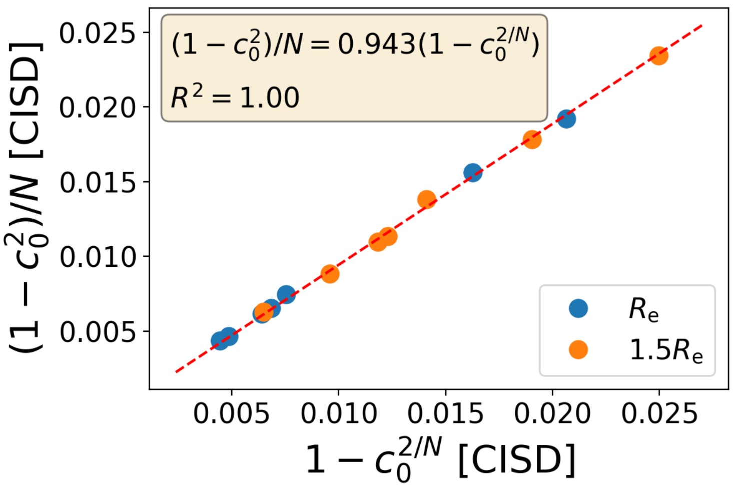

Figure S12: $\left(1-c_0^2\right)/N$ against $1-c_0^{2/N}$ for **Set A** including various diatomic molecules at equilibrium and 50% larger interatomic distances at the CISD level.

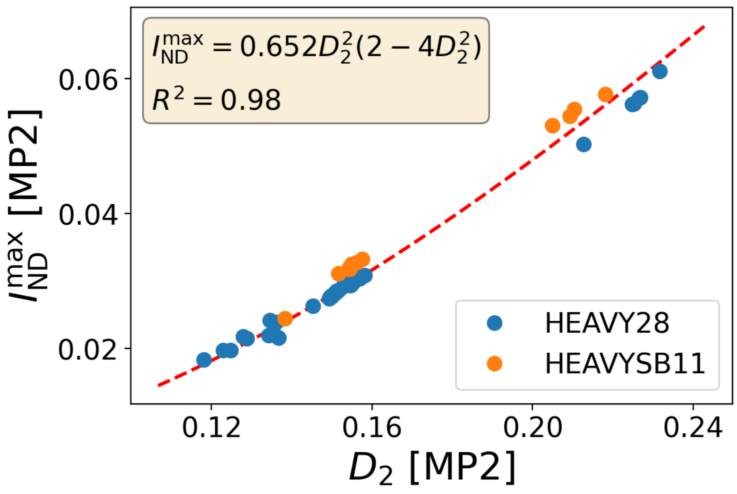

Figure S13: $I_{\mathrm{ND}}^{\mathrm{max}}$ against D_2 for the molecules in HEAVY28 and HEAVYSB11 datasets (both included in the GMTKN55, which is part of **Set D**).

Basis Set Dependence

Figures S14 and S15 study the dependency of the $\overline{I_D}$ and $\overline{I_{ND}}$ on the basis set. To this aim, we studied the molecules of set A at the MP2 level using 6-31G*, cc-pVDZ, cc-pVDT, aug-cc-pVDZ, aug-cc-pVTZ basis sets. For each basis set, we computed the relative deviation with respect to the average value, *i.e.*,

$$\langle \overline{I_{ND}} \rangle = \frac{1}{5}\sum_{k}^{\text{basis}} \overline{I_{ND}}_k \quad (1)$$

$$\%\text{Error}\left(\overline{I_{ND}}\right) = \frac{\left|\overline{I_{ND}} - \langle \overline{I_{ND}} \rangle\right|}{\langle \overline{I_{ND}} \rangle} 100\%, \quad (2)$$

and we can likewise define $\%\text{Error}\left(\overline{I_D}\right)$. Figure S14 shows that $\overline{I_D}$ has a homogeneous basis set dependence error, as expected for a dynamic correlation indicator, whereas $\overline{I_{ND}}$ shows a mild basis-set dependence.

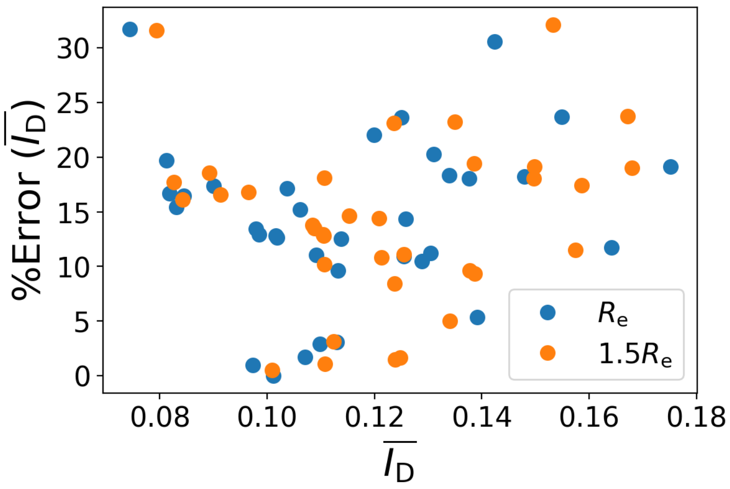

Figure S14: $\%\text{Error}\left(\overline{I_D}\right)$ against $\overline{I_D}$ for the molecules in **Set A**.

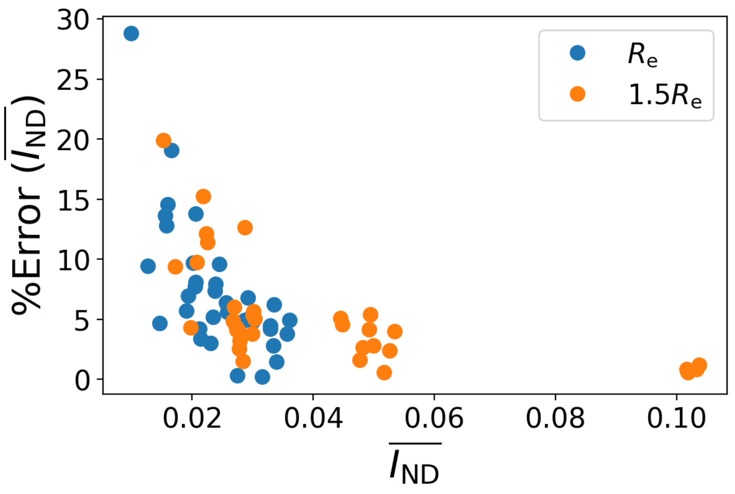

Figure S15: %Error $\left(\overline{I_{\mathrm{ND}}}\right)$ against $\overline{I_{\mathrm{ND}}}$ for the molecules in **Set A**.

References

(1) Turney, J. M.; Simmonett, A. C.; Parrish, R. M.; Hohenstein, E. G.; Evangelista, F. A.; Fermann, J. T.; Mintz, B. J.; Burns, L. A.; Wilke, J. J.; Abrams, M. L., et al. Psi4: an open-source ab initio electronic structure program. *WIREs, Comput. Mol. Sci.* **2012**, *2*, 556–565.

(2) Parrish, R. M.; Burns, L. A.; Smith, D. G.; Simmonett, A. C.; DePrince III, A. E.; Hohenstein, E. G.; Bozkaya, U.; Sokolov, A. Y.; Di Remigio, R.; Richard, R. M., et al. Psi4 1.1: An open-source electronic structure program emphasizing automation, advanced libraries, and interoperability. *J. Chem. Theory Comput.* **2017**, *13*, 3185–3197.

(3) Smith, D. G.; Burns, L. A.; Sirianni, D. A.; Nascimento, D. R.; Kumar, A.; James, A. M.; Schriber, J. B.; Zhang, T.; Zhang, B.; Abbott, A. S., et al. Psi4NumPy: An interactive quantum chemistry programming environment for reference implementations and rapid development. *J. Chem. Theory Comput.* **2018**, *14*, 3504–3511.

(4) Sharma, S.; Holmes, A. A.; Jeanmairet, G.; Alavi, A.; Umrigar, C. J. Semistochastic heat-bath configuration interaction method: Selected configuration interaction with semistochastic perturbation theory. *J. Chem. Theory Comput.* **2017**, *13*, 1595–1604.

(5) Smith, J. E.; Mussard, B.; Holmes, A. A.; Sharma, S. Cheap and near exact CASSCF with large active spaces. *J. Chem. Theory Comput.* **2017**, *13*, 5468–5478.

(6) Sun, Q.; Berkelbach, T. C.; Blunt, N. S.; Booth, G. H.; Guo, S.; Li, Z.; Liu, J.; McClain, J. D.; Sayfutyarova, E. R.; Sharma, S., et al. PySCF: the Python-based simulations of chemistry framework. *WIREs, Comput. Mol. Sci.* **2018**, *8*, e1340.

(7) Sun, Q.; Zhang, X.; Banerjee, S.; Bao, P.; Barbry, M.; Blunt, N. S.; Bogdanov, N. A.; Booth, G. H.; Chen, J.; Cui, Z.-H., et al. Recent developments in the PySCF program package. *J. Chem. Phys.* **2020**, *153*, 024109.

(8) Mok, D. K. W.; Neumann, R.; Handy, N. C. Dynamic and Nondynamic Correlation. *J. Phys. Chem.* **1996**, *100*, 6225–6230.

(9) Ramos-Cordoba, E.; Salvador, P.; Matito, E. Separation of dynamic and nondynamic correlation. *Phys. Chem. Chem. Phys.* **2016**, *18*, 24015–24023.

(10) Woon, D. E.; Dunning Jr, T. H. Gaussian basis sets for use in correlated molecular calculations. III. The atoms aluminum through argon. *J. Chem. Phys.* **1993**, *98*, 1358–1371.

(11) Crittenden, D. L.; Dumont, E. E.; Gill, P. M. Intracule functional models. II. Analytically integrable kernels. *J. Chem. Phys.* **2007**, *127*, 141103.

(12) Nielsen, I. M. B.; Janssen, C. L. Double-substitution-based diagnostics for coupled-cluster and Møller–Plesset perturbation theory. *Chem. Phys. Lett.* **1999**, *310*, 568–576.

(13) Goerigk, L.; Hansen, A.; Bauer, C.; Ehrlich, S.; Najibi, A.; Grimme, S. A look at the density functional theory zoo with the advanced GMTKN55 database for general main group thermochemistry, kinetics and noncovalent interactions. *Phys. Chem. Chem. Phys.* **2017**, *19*, 32184–32215.

(14) Duan, C.; Liu, F.; Nandy, A.; Kulik, H. J. Data-Driven Approaches Can Overcome the Cost–Accuracy Trade-Off in Multireference Diagnostics. *J. Chem. Theory Comput.* **2020**, *16*, 4373–4387.

(15) Dunning Jr, T. H. Gaussian basis sets for use in correlated molecular calculations. I. The atoms boron through neon and hydrogen. *J. Chem. Phys.* **1989**, *90*, 1007–1023.

(16) Kendall, R. A.; Dunning Jr, T. H.; Harrison, R. J. Electron affinities of the first-row atoms revisited. Systematic basis sets and wave functions. *J. Comput. Chem.* **1992**, *96*, 6796–6806.

(17) Sadlej, A. J. Medium-size polarized basis sets for high-level correlated calculations of molecular electric properties. *Collect. Czechoslov. Chem. Commun.* **1988**, *53*, 1995–2016.

(18) Nakano, M.; Kishi, R.; Nitta, T.; Kubo, T.; Nakasuji, K.; Kamada, K.; Ohta, K.; Champagne, B.; Botek, E.; Yamaguchi, K. Second hyperpolarizability (γ) of singlet diradical system: dependence of γ on the diradical character. *J. Phys. Chem. A* **2005**, *109*, 885–891.

(19) Hehre, W. J.; Ditchfield, R.; Pople, J. A. Self‚Äîconsistent molecular orbital methods. XII. Further extensions of Gaussian‚Äîtype basis sets for use in molecular orbital studies of organic molecules. *J. Chem. Phys.* **1972**, *56*, 2257–2261.

(20) Clark, T.; Chandrasekhar, J.; Spitznagel, G. W.; Schleyer, P. V. R. Efficient diffuse function-augmented basis sets for anion calculations. III. The 3-21+ G basis set for first-row elements, Li–F. *J. Comput. Chem.* **1983**, *4*, 294–301.

(21) Hurst, G. J.; Dupuis, M.; Clementi, E. A binitio analytic polarizability, first and second hyperpolarizabilities of large conjugated organic molecules: Applications to polyenes C_4H_6 to C_22H_24. *J. Chem. Phys.* **1988**, *89*, 385–395.

(22) Via-Nadal, M.; Rodríguez-Mayorga, M.; Ramos-Cordoba, E.; Matito, E. Singling Out Dynamic and Nondynamic Correlation. *J. Phys. Chem. Lett.* **2019**, *10*, 4032–4037.

(23) Ruedenberg, K.; Raffenetti, R.; Bardo, R. Energy, structure and reactivity. Proceedings of the 1972 Boulder Seminar Research Conference on Theoretical Chemistry. 1973; p 164.

(24) Feller, D. F.; Ruedenberg, K. Systematic approach to extended even-tempered orbital bases for atomic and molecular calculations. *Theor. Chim. Acta (Berlin)* **1979**, *52*, 231–251.

(25) Matito, E.; Cioslowski, J.; Vyboishchikov, S. F. Properties of harmonium atoms from FCI calculations: Calibration and benchmarks for the ground state of the two-electron species. *Phys. Chem. Chem. Phys.* **2010**, *12*, 6712.

(26) Ditchfield, R.; Hehre, W. J.; Pople, J. A. Self-consistent molecular-orbital methods. IX. An extended Gaussian-type basis for molecular-orbital studies of organic molecules. *J. Chem. Phys.* **1971**, *54*, 724–728.

(27) Bartlett, R. J.; Park, Y. C.; Bauman, N. P.; Melnichuk, A.; Ranasinghe, D.; Ravi, M.; Perera, A. Index of multi-determinantal and multi-reference character in coupled-cluster theory. *J. Chem. Phys.* **2020**, *153*, 234103.

(28) Dunning Jr, T. H. Gaussian basis functions for use in molecular calculations. I. Contraction of (9s5p) atomic basis sets for the first-row atoms. *J. Chem. Phys.* **1970**, *53*, 2823–2833.

(29) Karton, A.; Sylvetsky, N.; Martin, J. M. W4-17: A diverse and high-confidence dataset of atomization energies for benchmarking high-level electronic structure methods. *Journal of Computational Chemistry* **2017**, *38*, 2063–2075.

(30) Martin, J. M.; Santra, G.; Semidalas, E. An exchange-based diagnostic for static correlation. AIP Conf. Proc. 2022.

(31) Hobza, P. The calculation of intermolecular interaction energies. *Ann. Rep. Sec. C* **2011**, *107*, 148–168.

(32) Harris, C. R. et al. Array programming with NumPy. *Nature* **2020**, *585*, 357–362.

(33) Virtanen, P. et al. SciPy 1.0: Fundamental Algorithms for Scientific Computing in Python. *Nature Methods* **2020**, *17*, 261–272.

(34) pandas development team, T. Pandas-dev/pandas: Pandas. 2020; `https://doi.org/10.5281/zenodo.3509134`.

(35) Wes McKinney, Data Structures for Statistical Computing in Python. Proceedings of the 9th Python in Science Conference. 2010; pp 56 – 61.

(36) Hunter, J. D. Matplotlib: A 2D graphics environment. *Computing in Science & Engineering* **2007**, *9*, 90–95.

(37) Hellmann, H. *Einführung in die Quantenchemie*; Franz Deuticke: Leipzig and Vienna, 1937.

(38) Feynman, R. P. Forces in Molecules. *Phys. Rev.* **1939**, *56*, 340–343.

(39) Gordon, M. S.; Schmidt, M. W.; Chaban, G. M.; Glaesemann, K. R.; Stevens, W. J.; Gonzalez, C. A natural orbital diagnostic for multiconfigurational character in correlated wave functions. *J. Chem. Phys.* **1999**, *110*, 4199–4207.

(40) Matito, E.; Solà, M.; Salvador, P.; Duran, M. Electron sharing indexes at the correlated level. Application to aromaticity calculations. *Faraday Discuss.* **2007**, *135*, 325–345.
